# Public Health Surveillance After the 2010 Haiti Earthquake: the Experience of Médecins Sans Frontières

**DOI:** 10.1371/currents.dis.6aec18e84816c055b8c2a06456811c7a

**Published:** 2013-01-07

**Authors:** Jonathan Polonsky, Francisco Luquero, Gwenola Francois, Caroline Rousseau, Grazia Caleo, Iza Ciglenecki, Clara Delacre, M. Ruby Siddiqui, Mego Terzian, Leen Verhenne, Klaudia Porten, Francesco Checchi

**Affiliations:** Epicentre, Paris, France; Epicentre, Paris, France; Médecins Sans Frontières Operational Centre Paris, Paris, France; Médecins Sans Frontières Operational Centre Paris, Paris, France; Epicentre, Paris, France; Médecins Sans Frontières Operational Centre Geneva, Geneva, Switzerland; Médecins Sans Frontières Operational Centre Barcelona/Athens, Barcelona, Spain; Médecins Sans Frontières UK, London, United Kingdom; Médecins Sans Frontières Operational Centre Paris, Paris, France; Médecins Sans Frontières Operational Centre Brussels, Brussels, Belgium; Epicentre, Paris, France; Epicentre, Paris, France; Faculty of Infectious and Tropical Diseases, London School of Hygiene and Tropical Medicine, London, United Kingdom

## Abstract

Background In January 2010, Haiti was struck by a powerful earthquake, killing and wounding hundreds of thousands and leaving millions homeless. In order to better understand the severity of the crisis, and to provide early warning of epidemics or deteriorations in the health status of the population, Médecins Sans Frontières established surveillance for infections of epidemic potential and for death rates and malnutrition prevalence. Methods Trends in infections of epidemic potential were detected through passive surveillance at health facilities serving as sentinel sites. Active community surveillance of death rates and malnutrition prevalence was established through weekly home visits. Results There were 102,054 consultations at the 15 reporting sites during the 26 week period of operation. Acute respiratory infections, acute watery diarrhoea and malaria/fever of unknown origin accounted for the majority of proportional morbidity among the diseases under surveillance. Several alerts were triggered through the detection of immediately notifiable diseases and increasing trends in some conditions. Crude and under-5 death rates, and acute malnutrition prevalence, were below emergency thresholds. Conclusion Disease surveillance after disasters should include an alert and response component, requiring investment of resources in informal networks that improve sensitivity to alerts as well as on the more common systems of data collection, compilation and analysis. Information sharing between partners is necessary to strengthen early warning systems. Community-based surveillance of mortality and malnutrition is feasible but requires careful implementation and validation.

## Introduction

On 12^th^ January, 2010, Haiti was struck by a 7.0 magnitude earthquake which, according to Haitian authorities, killed over 200,000 people, made up to 1.5 million people homeless and resulted in massive economic and infrastructural damage.^1^ ^2^ Médecins Sans Frontières (MSF) mounted the largest relief operation in its history in response to the earthquake, establishing surgical, general medicine and mental health programmes in the affected areas, particularly within the capital Port-au-Prince.

In order to monitor health status in the areas of intervention and respond to emerging health threats, MSF established prospective surveillance to (i) detect and respond to outbreaks of epidemic-prone diseases based on observations from health facilities; and (ii) monitor mortality and nutritional status at the community level. This paper describes these surveillance systems, reports findings and discusses lessons learned from their implementation.

## Methods

**Surveillance of epidemic-prone diseases**

***Data collection***

Passive surveillance of suspected cases of epidemic-prone diseases was instituted as per standard protocol at all MSF-operated outpatient departments (OPDs), which served as sentinel sites. All five operational centres of MSF were involved in this system, under the coordination of an epidemiologist. The geographic coverage of these OPDs was considerable within Port-au-Prince, but low outside the capital (Table 1).

**Table 1: MSF reporting sites for post-earthquake surveillance of epidemic-prone diseases, Haiti 2010.** 

Reporting site	Location	**Week of first reporting**
Dufort	Dufort	6
Jacmel	Jacmel	6
Leogane	Leogane	7
Martissant	Port-au-Prince	3
Carrefour Feuille	Port-au-Prince	3
Choscal	Port-au-Prince	5
Grace	Port-au-Prince	6
Delmas 24	Port-au-Prince	6
Bicentennaire	Port-au-Prince	7
Champs de Mars	Port-au-Prince	7
Mickey	Port-au-Prince	7
Petionville	Port-au-Prince	9
Shekina	Port-au-Prince	9
St. Louis	Port-au-Prince	9
Sarthe	Port-au-Prince	13
Aviation	Port-au-Prince	21

Each OPD reported the number of consultations among children under 5 years old and among older persons due to any of the reportable diseases, with all other diagnoses grouped into the category "others" (Table 2). The frequency of reporting was initially daily at the request of the Ministère de la Santé Publique et de la Population (MSPP), and weekly starting in week 12. Reports from each OPD were consolidated into a central database. Coordination and sharing of urgent information was conducted by regular telephone contact with health facilities and their medical coordinators, and weekly meetings. Regular data and information on outbreak alerts were transmitted daily (subsequently weekly) to the Internally Displaced Persons Surveillance System (IDPSS) set up by the MSPP with the support of the United States Centers for Disease Control and Prevention (CDC). The IDPSS collated information from a larger number of reporting sites throughout Haiti, operated by various health agencies, of whom MSF was the largest in terms of geographic coverage and average number of sites reporting each week.

**Table 2: List of diseases under surveillance, MSF epidemic-prone disease surveillance system, Haiti 2010.** 

Condition	Case definitions
Acute respiratory infection	Fever over 38°C, with at least one of the following symptoms:Cough; sore throat; thoracic pain; respiratory complaints; general alteration of state.
Malaria	Fever during the last 24 hours plus Paracheck positive
Suspected case of cutaneous anthrax	Development of a papular-vesicular skin lesion, moving rapidly towards the bottom with black eschar and peripheral oedema (2-6 days), following contact with a sick animal.
Suspected case of pertussis	Irritating cough with inspiratory wheezing (whooping cough) of duration of at least one week. Often accompanied by expulsion of clear mucus and vomiting.
Acute watery diarrhoea	At least 3 non-bloody stools with abnormal consistency every 24 hours. May be accompanied by one or more of the following symptoms:Abdominal cramps; fever; vomiting.
Suspected case of typhoid fever	Fever over 39°C, lasting at least 3 days, with at least 2 of the following symptoms:Headache; abdominal pain; constipation or diarrhoea; vomiting; anorexia.
Acute bloody diarrhoea	At least 3 bloody stools every 24 hours. May be accompanied by one or more of the following symptoms:Abdominal cramps; fever; vomiting.
Suspected case of acute flaccid paralysis*	Relaxation of the muscles of the limbs with non-traumatic orgin, among children aged under 15 years.
Acute haemorrhagic fever syndrome*	Fever lasting between 2-7 days, accompanied by one or more of the following symptoms:Headache; retro-orbital pain; myalgia; arthralgia;and one or more of the following symptoms:Rash and bleeding tendencies; petechiae; bruising; purpura; mucosal bleeding; haematemesis or melaena; thrombocytopenia (100,000 platelets per mm3 or less); Ascites (plasma leakage).
Suspected case of dengue	Fever lasting between 2-7 days, accompanied by one or more of the following symptoms:Headache; retro-orbital pain; myalgia; arthralgia.
Febrile icteric syndrome	Fever and jaundice, with absence of predisposing factors, which can be accompanied by one or more of the following signs:Haemorrhagic rash or purpura; epistaxis; haemoptysis; other bleeding symptoms.
Suspected case of meningococcal meningitis *	Sudden onset of fever and stiff neck, with bulging fontanel among children aged under one year.Probable case: more turbid cerebro-spinal fluid or an epidemic in progress.
Suspected case of diphtheria*	Acute pharyngitis or laryngitis, with one or more grey false membranes, accompanied by swelling of the neck and obstruction of the airways.
Suspected case of measles*	Fever with non-pruritic maculopapular rash, with at least one of the following signs:Conjunctivitis ; rhinitis ; cough (oculo-nasal catarrh).
Suspected case of tetanus	Painful muscular contractions, generalised spasms, stiffness, trismus, with history of injury or evidence of an entrance of infection.
Bite by suspected rabid animal *	History of bite by suspected rabid animal, breathing difficulties, gasping interspersed with sighs and pauses, and hydrophobic spasms.

*Diseases requiring immediate notification

The system was in operation from week 3, 2010, the week following the earthquake, until week 31, 2010. The conditions under surveillance and their standard case definitions are shown in Table 2. This list was generated during discussions between IDPSS partner organisations, led by the MSPP.

**Data analysis & reporting**

Some conditions required immediate notification upon detection of a suspected case. In such instances, OPD staff were instructed to telephone both the Laboratoire National de la Santé Publique (LNSP) for collection of samples for confirmation, and the coordinating epidemiologist, who relayed this information among MSF sections and, together with the relevant medical coordinator, coordinated further investigations when warranted.

Aggregate case data were analysed using Microsoft Excel. A bulletin was created for each reporting period providing an overview of the situation, with special reference to suspected cases of conditions requiring immediate notification, and to changing trends in any condition under surveillance. While a single case of any immediately reportable condition was sufficient to generate an alert, for other, more frequent conditions (e.g. acute respiratory infection) we plotted trends in incident cases and proportional morbidity so as to detect important changes. No formal alert or outbreak thresholds were specified for these conditions, and we were unable to calculate incidence rates, as the total population in the catchment areas of each OPD was unknown and subject to substantial and rapid change (see Figures 7 and 8).

Bulletins were discussed with medical coordinators of each section and shared in the daily IDPSS meetings.

**Surveillance of mortality and malnutrition**

**Data collection**

Surveillance of death rates and malnutrition prevalence was community-based and exhaustive within two specific sites served by MSF-France: Delmas24, a neighbourhood of Port-au-Prince consisting of mixed residential layout (houses, tents, and small camps) and Champs de Mars, a dense community of displaced persons.

Surveillance of Delmas 24 was established by week 12, while surveillance of Champs de Mars was established by week 17, six and three weeks after MSF-France began operating these OPDs, respectively.

In each site, during the first week of data collection, the baseline size of the population under surveillance was estimated. In Delmas24, a team of home visitors counted all households, while an estimate of mean household size, necessary to compute the total population, was obtained through a stratified cluster survey, which was also used to assess basic needs (results not shown). In Champs de Mars, home visitors did an exhaustive census. Information on measles and DTP3 vaccination coverage, and awareness of MSF activities, was also collected during the census by interviewing a systematic sample of households (results not shown).

Subsequently, each site was apportioned approximately equally among home visitors (with 24 sectors in Delmas 24 and 20 in Champs de Mars). All households under surveillance were visited once each week, between Monday and Saturday, and information was collected on births, deaths and movements into and out of households. We also attempted to collect census information for newly-established households and those households which had left or ceased existing since the previous week. This information was used to update the baseline population size and compute crude and under 5y death rates. In addition, home visitors assessed the prevalence of acute malnutrition among children aged 6-59 months by mid-upper arm circumference (MUAC) and bilateral oedema measurement. For sample size considerations, these nutritional assessments were done in a systematic random sample of households (every 5^th^ household, changing each week), in which all children aged 6-59 months living in the selected households were assessed. Finally, information on the treatment history of decedents and on the enrolment of malnourished children within nutritional programmes was also collected, for use as a proxy measure of coverage of MSF's interventions.

The system in both sites was in operation until week 22, when reports of threats against home visitors forced its closure.

**Data analysis & reporting**

Data were entered in EpiData (EpiData Association, Odense, Denmark) between Monday and Wednesday of the week following data collection, and analysed using Microsoft Excel, with a short bulletin written each Thursday. Bulletins reported crude and under-5y death rates (CDR and U5DR, respectively) per 10,000 person-days, as well as estimates of severe (MUAC < 115mm and/or bilateral oedema) and global (MUAC <125 mm and/or bilateral oedema) acute malnutrition (SAM and GAM, respectively) among children aged 6-59 months, and descriptions of population movements.

## Results

**Surveillance of epidemic-prone diseases**

The number of cases of, and proportional morbidity due to, each condition under surveillance are presented in Table 3.

**Table 3: Cumulative number of cases of diseases under surveillance, by age group, and proportional morbidity, MSF surveillance system, Haiti, 2010.** 

Disease	<5 years	≥5 years	Total Cases	Proportional morbidity (%)
Acute diarrhoea (watery)	4,262	1,601	5,863	5.7
Acute diarrhoea (bloody)	207	152	359	0.4
Acute respiratory infection	12,852	13,818	26,670	26.1
Malaria/ Fever of unknown origin	939	3,644	4,603	4.5
Dengue	-	1	1	0.0
Acute jaundice	11	69	80	0.1
Typhoid (suspected)	65	391	456	0.4
Measles (suspected)	4	3	7	0.0
Meningitis (suspected)	11	1	12	0.0
Diphtheria (suspected)	1	1	2	0.0
Pertussis	-	-	-	-
Tetanus	2	1	3	0.0
Neonatal tetanus	-	-	-	-
Tuberculosis (suspected)	8	72	80	0.1
Acute flaccid paralysis	-	2	2	0.0
Acute haemorrhagic fever	-	-	-	-
Others	8,734	55,182	63,916	62.6
**Total**	**27,096**	**74,958**	**102,054**	**100.0**

Overall, there were 102,054 consultations at the 16 reporting sites during the 26 weeks the surveillance system was in operation. The majority of these (62.6%) had a diagnosis of "other". Acute respiratory infections (ARIs) accounted for a quarter of the overall proportional morbidity and approximately half among children aged under 5y. Other leading causes of consultation were acute watery diarrhoea (AWD) and malaria/fever of unknown origin (FUO).

While the number of cases of AWD and malaria/FUO remained relatively stable, cases of ARI increased throughout the reporting period (Figure 1). On average, completeness of health facility reporting was above 80%.

**Trends in numbers of suspected cases of selected syndromes (left axis) and completeness of reporting (right axis), MSF disease surveillance system, Haiti, 2010.**
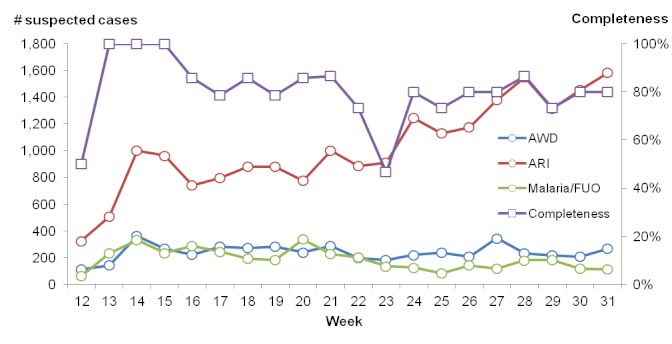

During its period of operation, the system generated several alerts due to various suspected diseases. One was triggered by the detection of a cluster of acute onset jaundice in Leogane, suspected to be leptospirosis. MSF immediately notified the LNSP, who launched an investigation, and the aetiology was confirmed as Hepatitis A in those cases that were able to be traced. No further cases were noted. Another was triggered by the detection of a single dengue case, but no additional cases were noted thereafter. There were several alerts triggered by suspected measles cases, but in all instances, the laboratory results were negative for measles.

The final alert was triggered when the reporting focal point for an MSF section reported observing an increase in the number of suspected typhoid cases. Between week 13 and week22, a total of 208 suspected cases of typhoid were seen at OPD Choscal, based in the slum district of Cité Soleil, with a peak of 39 cases presenting in week 17 (Figure 2).

**Date of onset of symptoms among Typhoid cases (suspected and confirmed), Choscal hospital, 2nd – 14th March 2010 (n=50).**
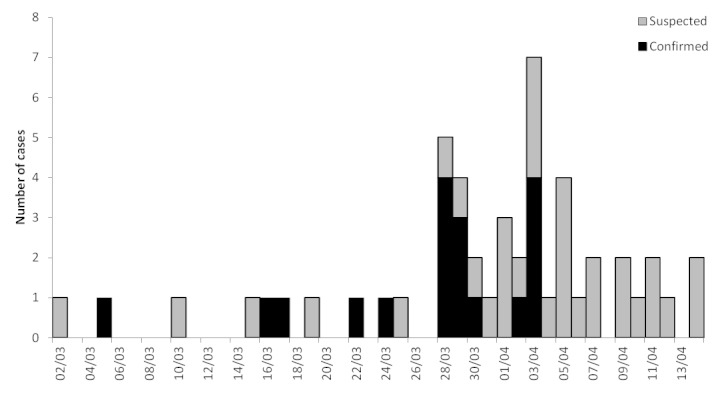

An investigation was launched, in which inpatients or their caregivers were interviewed about water usage, and a common water source (Chateau d’eau, a water resevoir) was directly implicated in the majority (70%) of cases. Laboratory tests conducted by the ‘Centrale Autonome Métropolitaine d’Eau Potable’ at the source, which had been structurally damaged during the earthquake, showed the presence of dangerous levels of faecal and other pathogenic bacteria. The water was subsequently treated, which coincided with a subsiding of the outbreak.

**Surveillance of mortality and malnutrition**

The results of the surveillance system are presented in figures 3-6.

**Trends in crude (CDR) and under-5 (U5DR) death rates, Delmas 24, Port-au-Prince, Haïti, weeks 13 – 22, 2010.**
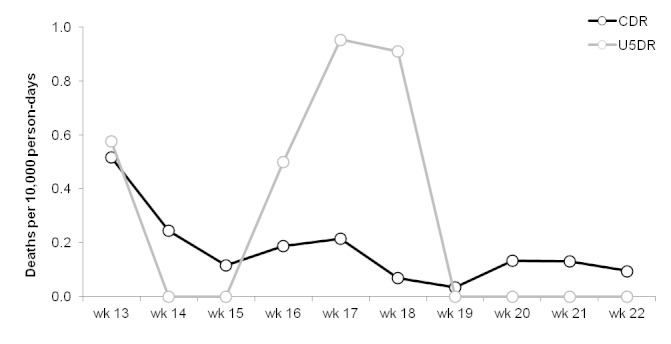

**Trends in crude (CDR) and under-5 (U5DR) rates, Champs de Mars, Port-au-Prince, Haïti, weeks 17 – 22, 2010.**
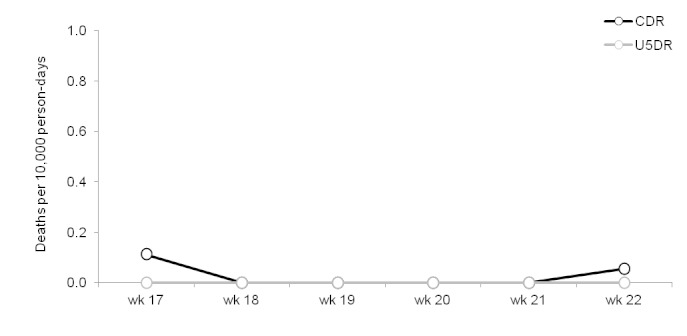

**Trends in severe (SAM) and global (GAM) acute malnutrition prevalence, Delmas 24, Port-au-Prince, Haiti, weeks 13 – 22, 2010. Vertical bars indicate 95% confidence intervals.**
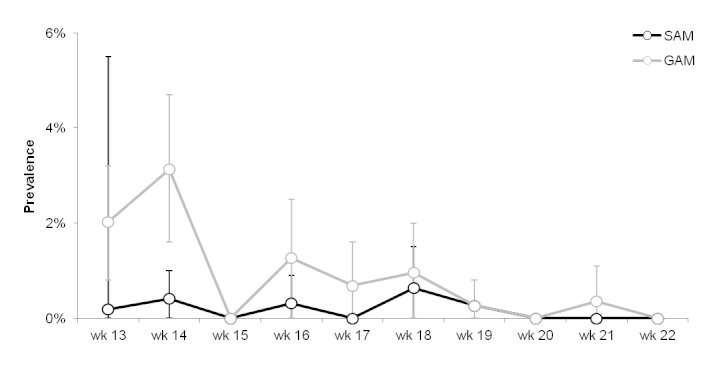

**Trends in severe (SAM) and global (GAM) acute malnutrition prevalence, Champs de Mars, Port-au-Prince, Haiti, weeks 17 – 22, 2010. Vertical bars indicate 95% confidence intervals.**
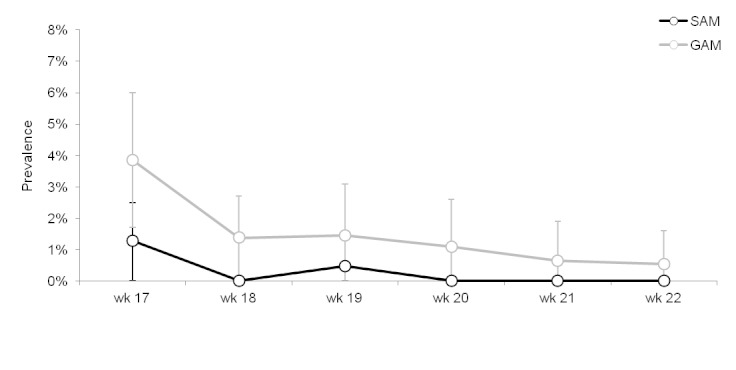

CDR and U5DR in both sites were generally below established emergency thresholds for Latin America and the Caribbean (≥0.3 deaths per 10,000 person-days for both CDR and U5DR) throughout the period of surveillance.^3^ In Delmas 24, both CDR and U5DR were above the emergency threshold during the first week of data collection, and U5DR was above this threshold during 3 subsequent weeks.

The prevalence of both SAM and GAM was highest in the weeks during which the system was being established and thereafter gradually declined. These prevalences were below alarm thresholds (SAM ≥3%; GAM≥10%).^3^

In Delmas 24 there was a substantial net increase in the size of the population under surveillance, from approximately 43,930 (95% confidence interval (CI): 42,746-45,093) at baseline to 54,894 at the time the surveillance system was ended, an increase of 25.0% (Figures 7 and 8).

**Weekly arrivals and departures of people (left axis) and total population (right axis), Delmas 24, Port-au-Prince, Haiti, weeks 13 – 22, 2010.**
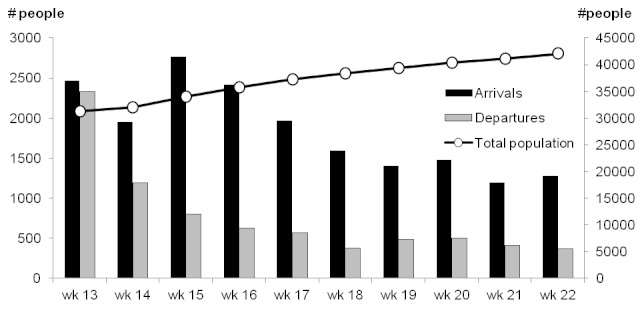

**Weekly arrivals and departures of people (left axis) and total population (right axis), Champs de Mars, Port-au-Prince, Haiti, weeks 17 – 22, 2010.**
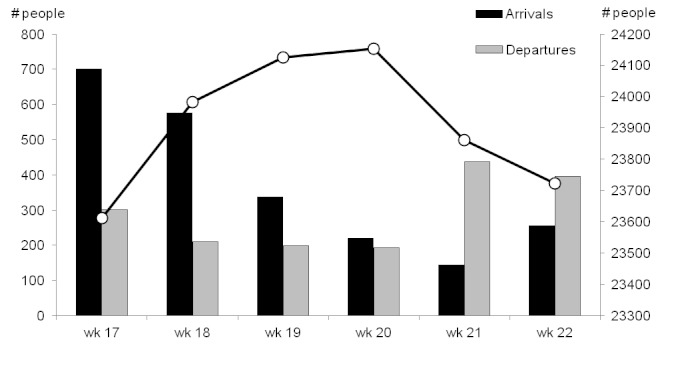

A discrepancy in population estimations was apparent; according to the initial survey-derived population estimation, followed by weekly population movement tracking, the total population under surveillance was52,202 in week 19. However, an exhaustive census conducted during the same week reported a population of 39,349, a difference of -32.7%.

In Champs de Mars, after an initial increase, the size of the population under surveillance decreased due to the departure of many households, who were at the time under threat of expulsion from the occupied public space.

## Discussion

The surveillance system for epidemic-prone diseases involved coordination of five MSF sections and provided the majority of information to a country-wide system operated by the MSPP. Furthermore, it was implemented with minimal delay following the earthquake, despite difficult circumstances.

One important difficulty was that implementation of uniform case definitions at the health clinic level was problematic, particularly where the MSPP case definitions did not match those routinely used by different MSF sections.

The major limitation of the system, at least initially, was that it placed a considerable additional burden of reporting on health staff. The process of acquiring data and compiling reports each day was felt to be overly time-consuming, and there was wide variation in the timeliness and completeness of reporting between sites. The change to weekly reporting reduced workload and improved completeness and timeliness of reporting and was not judged to impair the system. Indeed, the benefit of daily incidence reporting is not clear when there is no outbreak suspected or confirmed, particularly since the system already required immediate notification of infections of epidemic potential.

Excessive focus on data reporting, transmission and management may withdraw resources from other important functions of any surveillance system, notably creation of a network of information providers, reinforcement of alert procedures, in-service training on case definitions and reporting procedures, and response to alerts generated. Evidence suggests that the vast majority of alerts and outbreaks are not detected through trends analysis on aggregate data reports, but rather through formal or informal notifications by health facilities and other informants.^4^ ^5^ Although the centralised inter-sectional system relied exclusively upon sentinel site data, each sentinel site analysed their own data and set up informal information networks, including regular meetings with key informants and community representatives, as is common practise. Each site was therefore positioned to react to information and alerts independently, without necessarily waiting for alerts to come through the centralised system.

Sources of data are an important consideration when establishing a surveillance system. We relied upon OPDs acting as sentinel sites, resulting in a system with high sensitivity, but probably low specificity, due to the fact that people present at OPDs for a variety of complaints, the majority of which are not relevant for surveillance of epidemic-prone diseases. Where these have been established, inpatient departments, which tend to receive more severely ill patients, could serve as an additional source of data. This has the effect of screening out a significant proportion of the ‘background noise’ of people presenting at OPDs for mild conditions, thereby increasing the specificity of the surveillance system. However, this does not address the inherent bias towards the most ill, those with no barriers to access, and those who do not seek alternative health care in an exclusively facility-based surveillance system.

A striking finding was that approximately two-thirds (62.6%) of conditions for which people presented were listed as ‘other’. This high proportion is due principally to the high proportion of consultations for chronic and other conditions not included in the list of diseases under surveillance. In the lifetime of this system, these were primarily related to mental health and physical injuries sustained during the earthquake, as well as typical chronic conditions such as cardiovascular disease and diabetes.

As is the common experience, ARIs, AWD and malaria/FUO were predominant causes of morbidity. It should be noted that FUO may be due to causes other than malaria, particularly as malaria incidence is low in Haiti. The steady increase in weekly reported cases of ARI, from506 in week 13 to1,584 in week 31, was not detected at the time and did not trigger a formal alert, suggesting that this surveillance system was more sensitive to sudden onset outbreaks in immediately notifiable diseases, such as suspected cases of measles and leptospirosis, and in diseases with relatively high case fatality ratios (CFR), such as typhoid. Greater emphasis should be placed on detecting and responding to slower, steady increases in diseases with lower CFR such as ARI which nevertheless account for large proportional morbidity and mortality due to their high incidence.^6^

The detection, investigation and verification of rumours or other alerts of suspicious episodes and clusters of disease is increasingly recommended as a surveillance tool in humanitarian emergencies, based on recent experiences and recommendations.^4^ ^7^ It offers a light-weight complementary system for the detection of a variety of conditions, both anticipated and unanticipated, with a reduction in workload. However, the sensitivity of this methodology is unclear, and dependent on a variety of factors. Crucially, excellent communication links with both peripheral health facilities and the communities at risk must be in place in order to detect rumours and alerts.

Finally, while geographic coverage of the system was high within Port-au-Prince, the number of sentinel sites elsewhere was low. To some extent, this was justified on the basis of where most damage and displacement had occurred.^8^ However, it arguably also reflected the tendency of relief agencies to focus on the more visibly stricken, easily accessible urban communities. While rural areas were less affected by the earthquake itself, they received large numbers of IDPs in subsequent months. Indeed, the outbreak of cholera which began in October 2010 started in a rural area, highlighting the importance of ensuring any disease surveillance system has adequate geographic coverage.^2^ ^9^

Critically, the list of reportable conditions in our surveillance system, as per the country-wide IDPSS, did not include suspected cholera, based on a risk assessment that there was low risk from this pathogen in Haiti,^10^ although some MSF sections had included this disease within their own surveillance activities. Accordingly, epidemic preparedness measures also excluded cholera. The 2010 Haiti cholera epidemic (which began after the closure of our surveillance system) suggests a need for better epidemic risk assessments in future.

Estimates of mortality and malnutrition prevalence were lacking during the weeks after the earthquake, but a common assumption was that mortality was very high, possibly at emergency levels. Our results were not generalisable beyond the areas included, but did reflect conditions in two of the most severely affected areas in Port-au-Prince. As such, this community-based system was judged to be of considerable value to MSF, and attracted interest from a variety of public health actors including the MSPP, as it shifted focus away from reducing presumed high mortality and malnutrition to other activities such as distribution of essential non-food items and chronic disease case.

Although death rates were relatively low and stable during the period of surveillance, the U5DR in Delmas 24 did cross the emergency threshold for the context on three occasions. Notably, just one death among the under-5 year-olds was sufficient to produce a greater-than emergency-level death rate of 0.5 deaths per 10,000 person-days in Delmas 24 during week 16. Similarly, a single death among those aged under 5 would have had the same effect in Champs de Mars. This demonstrates an important feature of such surveillance systems, namely that the results may only be meaningful if the denominator is of a sufficient size, and that analysing trends over time is essential. In this case, aggregating the data by month rather than by week, or presenting moving averages, were advisable alternatives.

Prevalence of malnutrition was low and stable during the period of surveillance, providing useful information on the absence of a nutritional crisis at a time when such information was unknown. The decline in malnutrition prevalence after the initial weeks may be a result of the detection of a large proportion of the prevalent malnourished children during the first few weeks, subsequent to which only the incident cases were detected. There may also have been some measurement biases (e.g. inaccurate anthropometric measurements) associated with the establishment of the system.

In addition to the weekly updates on mortality and malnutrition, and the results of one-off surveys on basic needs and vaccination coverage, the system raised awareness about the presence of local MSF OPDs and provided regular access to the intended beneficiaries of MSF's programmes. Each week, home visitors relayed messages and feedback from the population, helping MSF to understand better their needs and concerns. This proximity also had the potential to feed into disease surveillance, as MSF kept an ‘ear-to-the-ground’ for detecting rumours of strange disease clusters, although no such rumours were reported during the period the system was in operation.

Threats against the home visitors which forced the systems' premature closure revealed an important lesson. During weekly visits, home visitors reminded households that MSF was providing free-of-charge health care in the area, and MSF also carried out large distributions of non-food items in the sites. However, home visitors reported on community members' frustration that, despite weekly visits, surveillance yielded no immediate, tangible benefit for the population. Further consideration is needed to understand the causes of frustration for populations under surveillance, and how best to address or avoid aggravating these sentiments, e.g. reducing the frequency of home visits to once per month.

The very low levels of mortality detected in Champs de Mars are suggestive of under-detection of a portion of deaths. A similar system, established by Epicentre and MSF-Spain in rural Central African Republic, was shown to have high sensitivity for mortality.^11^ In that study, concerns arose regarding the reliability of population estimates: the population size discrepancies noted in this system in Delmas 24 suggest similar problems, which might affect the estimates of death rates obtained. However, the population movements described do fit the patterns that were known to have occurred in both areas. We could not determine whether bias lay mainly in the baseline estimate, the subsequent census, weekly surveillance, or some combination of these.

## Conclusions

The duplication of efforts in the compilation and analysis of disease surveillance data was time consuming, burdensome and of minimal added value, particularly during the daily reporting phase which coincided with the acute phase of the emergency. However, the alert and response component was established early and functioned well, and disease surveillance after disasters should always include such components. Concretely, this means investing time and resources in the informal networks that can detect rumours and other alerts and the laboratory network and transport of samples for verification of immediately notifiable diseases, as well as on the systems of data collection, compilation and analysis more commonly in place. Information must be shared regularly with partners to strengthen the early warning component of disease surveillance in crises, which should require the immediate notification of diseases of epidemic potential.

The experience with community surveillance demonstrates that such systems are feasible but require careful implementation, including quick exercises to validate the mortality estimates produced and periodic repeat censuses, to ensure an accurate denominator is used to obtain rates, to prevent biased estimates and to increase community acceptance.

## Competing Interests

The authors have declared that no competing interests exist.

## Correspondence

Jonathan Polonsky. Email: jonathan.polonsky@epicentre.msf.org
